# Occupational noise-induced hearing loss in India

**DOI:** 10.4103/0019-5278.43260

**Published:** 2008-08

**Authors:** Subroto S. Nandi, Sarang V. Dhatrak

**Affiliations:** National Institute of Miners’ Health, JNARDDC Campus, Wadi, Nagpur - 440 023, India

**Keywords:** Audiogram, compensation, hearing protectors, noise-induced hearing loss, occupational noise, prevalence

## Abstract

Noise is the insidious of all industrial pollutants, involving every industry and causing severe hearing loss in every country in the world. Exposure to excessive noise is the major avoidable cause of permanent hearing impairment. Worldwide, 16% of the disabling hearing loss in adults is attributed to occupational noise, ranging from 7 to 21% in the various subregions. The estimated cost of noise to developed countries ranges from 0.2 to 2% of the gross domestic product (GDP). Noise-induced hearing loss (NIHL) is bilateral and symmetrical, usually affecting the higher frequencies (3k, 4k or 6k Hz) and then spreading to the lower frequencies (0.5k, 1k or 2k Hz). Other major health effects are lack of concentration, irritation, fatigue, headache, sleep disturbances, etc. The major industries responsible for excessive noise and exposing workers to hazardous levels of noise are textile, printing, saw mills, mining, etc. Hearing protectors should be used when engineering controls and work practices are not feasible for reducing noise exposure to safe levels. Earmuffs, ear plugs and ear canal caps are the main types of hearing protectors. In India, NIHL has been a compensable disease since 1948. It is only in 1996 that the first case got compensation. Awareness should be created among workers about the harmful effects of noise on hearing and other body systems by implementing compulsory education and training programs. There are very few published studies of NIHL in India. More extensive studies are needed to know the exact prevalence of NIHL among the various industries in India.

## INTRODUCTION

Noise is the insidious of all industrial pollutants, involving every industry and causing severe hearing loss in every country in the world. Occupational hearing loss includes acoustic traumatic injury and noise-induced hearing loss (NIHL), and can be defined as a partial or complete hearing loss in one or both ears as the result of ones employment. Exposure to excessive noise is the major avoidable cause of permanent hearing impairment worldwide. NIHL is an important public health priority because as populations live longer and industrialization spreads, NIHL will add substantially to the global burden of disability. In many countries, excessive noise is the biggest compensatable occupational hazard. Worldwide, 16% of the disabling hearing loss in adults is attributed to occupational noise, ranging from 7 to 21% in the various subregions.[1] The estimated cost of noise to developed countries ranges from 0.2 to 2% of the GDP, where it is the cause of more than one-third of the hearing impairments. The effects of the exposure to occupational noise are higher in the developing regions.[2] There is a lack of epidemiological data on prevalence, risk factors and costs of NIHL in India. In this article, we have attempted to review the available occupational NIHL problem in India.

## NOISE-INDUCED HEARING LOSS

NIHL is generally used to denote the cumulative, permanent loss of hearing that develops gradually after months or years of exposure to high levels of noise. It has long been recognized as a problem in occupations associated with prominent noise. NIHL is the second most common form of acquired hearing loss after age-related loss (presbycusis), with studies showing that people who are exposed to noise levels higher than 85 db suffered from NIHL.[3] A typical NIHL is of a sensory neural type involving injury to the inner ear. It is bilateral and symmetrical, usually affecting the higher frequencies (3k, 4k or 6k Hz) and then spreading to the lower frequencies (0.5k, 1k or 2k Hz).[4] Impairment of hearing at high frequencies will initially cause a loss of clarity in perceived speech and then interfere with daily activities as hearing loss progresses. Hearing loss-related symptoms, such as trouble in normal and telephone conversation, turning up the radio/television volume and tinnitus, usually occur in the early stages of NIHL.[5] Other major health effects due to the noise pollution are lack of concentration, irritation, fatigue, headache, sleep disturbances, etc. The risk of hearing loss and injury to the ears increases with the sound intensity, the length of time an employee is exposed to noise and the individual susceptibility to NIHL.

## PATHOGENESIS OF NOISE-INDUCED HEARING LOSS

**Figure 1:**
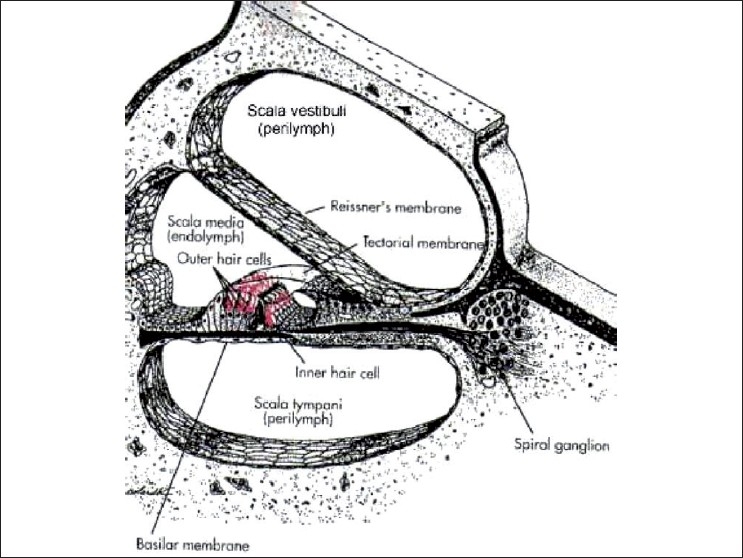
A cross section of the human cochlea

Sound travels from external ear and falls on the tympanic membrane, which is then set into vibration and these vibrations are transmitted to the middle ear where the sensory hair cells in the cochlea are responsible for initiating the neural impulses that carry information to the brain regarding the sounds. The human cochlea has one row of inner hair cells and three rows of outer hair cells [Figure 1].[6] The outer rows of hair cells run throughout the length of the cochlea. The hair cells responding to higher frequency sounds are located closer to the basal end of the cochlea, and those most sensitive to lower frequency sounds are found toward the apical end of cochlea. The amount and the type of direct hair cell damage depends on the intensity of the sound. Exposure to noise at subtraumatic level exhibits a temporary shift in hearing sensitivity that returns to normal with time away from the hazardous exposure. However, higher sound levels damage the outer hair cells, stereocilia, further, including destruction of the intercilial bridges, and recovery takes longer. An even higher level of sound leads to a collapse of the stereocilia and the hair cell is eventually damaged permanently. If the outer hair cells are not functioning, a greater stimulation is required to initiate a nervous impulse; thus, the threshold sensitivity of inner hair cells is raised, which is perceived as a hearing loss. Once damaged, the auditory sensory cells cannot repair themselves nor can the medical procedures restore normal function.[7]

## OCCUPATIONS AT RISK

In India, occupational permissible exposure limit for 8 h time weighted average is 90 dBA.[8] The major industries responsible for excessive noise and exposing workers to hazardous levels of noise are textile, printing, saw mills, mining, etc. Studies carried out by the National Institute of Occupational Health, India, showed that the sound pressure levels were very high in various industries of India.[9]

The noise levels in different industries is given in Table 1.

**Table 1:** Noise levels in different industries

Industries	Range (dBA)
Textile industries	102-114
Pharmaceutical firms	93-103
Fertilizer plants	90-102
Oil and natural gas complex in Bombay high	90-119
Road traffic in Ahmedabad city	60-102
Surface rail traffic	90-102
Metro rail	70-111
Air traffic	90-112

## DIAGNOSIS OF NIHL

Audiometry is a standard test to detect and evaluate hearing loss. Audiometry is used to determine the auditory threshold of an individual to pure tones of 250-8000 Hz and sound levels between 10 (the hearing threshold of intact ears) and 110 dB (maximal damage). The patient should not have been exposed to noise during the previous 16 h to eliminate the effects of a temporary threshold shift. Air conduction is measured by ear phones placed on the ears, while bone conduction is measured by placing a vibrator in contact with the skull behind the ears. Each ear is evaluated separately and test results are reported on a graph known as an audiogram. Comparison of air and bone conduction allows classification of hearing loss as conductive or sensorineural. The audiogram in case of NIHL is characterized by an onset of hearing loss at 4000 Hz, visible as a dip in the audiogram. As exposure to excessive noise level continues, neighboring frequencies are progressively affected and the dip broadens, intruding into neighboring frequencies. NIHL is usually bilateral and shows a similar pattern in both the ears. The difference between the two ears should not exceed 15 dB at 500, 1000 and 2000 Hz and 30 dB at 3000, 4000 and 6000 Hz, respectively.[7]

## GRADING OF THE HEARING IMPAIRMENT

The WHO recommended the following classification on the basis of the pure tone audiogram taking the average of the thresholds of hearing for frequencies of 500, 1000, 2000 and 4000 Hz.[10] Table 2 shows the grading of hearing impairment.

**Table 2:** Grading of the hearing impairment

Grade of impairment	Corresponding audiometric ISO value	Performance	Recommendations
0 - No impairment	25 dB or better (better ear)	No or very slight hearing problems. Able to hear whispers	
1 - Slight impairment	26-40 dB (better ear)	Able to hear and repeat words spoken in normal voice at 1 m	Counseling. Hearing aids may be needed
2 - Moderate impairment	41-60 dB (better ear)	Able to hear and repeat words spoken in a raised voice at 1 m	Hearing aids usually recommended
3 - Severe impairment	61-80 dB (better ear)	Able to hear some words when shouted into the better ear	Hearing aids needed. If no hearing aids available, lipreading and signing should be taught
4 - Profound impairment including deafness	81 dB or greater (better ear)	Unable to hear and understand even a shouted voice	Hearing aids may help understand words. Additional rehabilitation needed. Lipreading and sometimes signing essential

Grades 2, 3 and 4 are classified as disabling hearing impairment. The audiometric ISO values are averages of values at 500, 1000, 2000 and 4000 Hz

## CALCULATION OF THE HEARING IMPAIRMENT

To express the hearing impairment in terms of percentage, different countries and professional bodies have adopted their own system to calculate the percentage. One of the methods to find hearing impairment is as follows:

1. From the audiogram, calculate the average of the thresholds of hearing for frequencies of 500, 1000, 2000, 4000 and 6000 Hz.
2. Deduct from it 25 dB (as there is no impairment up to 25 dB).
3. Multiply it by 1.5.

This is the percentage of hearing impairment for that ear. Similarly, calculate the percentage of hearing impairment for the other ear. The percentage handicap of an individual is calculated using the formula given below.[7]

$$\text{Percentage handicap of an individual}=\frac{\left(\text{Better ear}\%\times \text{5}\right)+\left(\text{worse ear}\%\right)}{6}$$

## OCCUPATIONAL NIHL

The published studies of NIHL in India are limited, which are cited here. In a cross-sectional study, the hearing status of tractor-driving farmers (TDFs) was compared with that of non-TDFs. All participants were interviewed for details of work routine and noise exposures. Audiogram analysis showed higher prevalence of abnormalities in TDFs. TDFs more often had a higher frequency of hearing loss when compared with non-TDFs.[11]

A study was conducted in heavy engineering industry, which included machines shop and press divisions. The sound levels ranged from 83 to 116 dBA. Hearing impairment was progressive in all the study groups.[12]

In a textile mill weavers study, the sound levels were around 102-104 dBA and the hearing acuity of the textile weavers was found to be poor. NIHL at 4000 Hz was as high as 30 dB in the age range 25-29 years, 40 dB in the age range 30-34 years and 45 dB in the age range 35-39 years.[13]

A study was conducted in a drug and pharmaceutical company where the noise levels were 100-105 dBA. Significant NIHL was found in the workers.[14]

A survey on the effects of noise pollution on traffic policemen in the city of Hyderabad, India, carried out by the Society to Aid the Hearing Impaired, revealed that 76% had NIHL. Among these, all those who had completed 5 years in the traffic wing had hearing loss in various degrees.[15]

The National institute of miners’ health (NIMH) has carried out NIHL studies in various mines. NIHL was prevalent among 12.8% of the employees. Moderate NIHL was detected in 10.2% and severe NIHL was observed in 2.6% of the employees.[16]

There are other industries like construction, printing, saw mills and crushers where the workers are exposed to high levels of noise throughout their lifetime of work. Studies regarding NIHL among the workers of these industries are not available.

## COMPENSATION

In India, NIHL has been a compensable disease since 1948 under the Employees State Insurance Act (1948) and the Workmen's Compensation Act (1923). But still there is very little awareness regarding this fact. Nearly 3 billion dollars has been paid as compensation for NIHL in the USA in the last two decades. In India, it was only in 1996 that the first case got compensation, and about 250 workers are receiving compensation for NIHL.[17]

## EAR PROTECTION AND CONSERVATION

The most effective way to prevent NIHL is to protect the worker from hazardous noise at the workplace. Hearing protectors should be used when engineering controls and work practices are not feasible for reducing noise exposure to safe levels. A personal hearing protection device is a device designed to reduce the level of sound reaching the eardrum. Ear muffs, ear plugs and ear canal caps are the main types of hearing protectors.[7] To select hearing protectors, we should consider the following:

1. The workers who will be wearing them.
2. The need for compatibility with other safety equipment.
3. Workplace conditions such as temperature, humidity and atmospheric pressure.

A variety of style should be provided so that workers may select a hearing protector on the basis of comfort, ease of use and handling and impact on communication. Each worker should receive individual training in the selection, fitting, use, repair and replacement of hearing protectors. The most common excuses reported by workers for not wearing hearing protectors include discomfort, interference with hearing speech and warning signals and the belief of workers that there is no control over an inevitable process that causes hearing loss.[18] Given adequate education and training, workers can realize the crucial importance of wearing hearing protectors.

## CONCLUSION

Noise is the hazardous industrial pollutant causing severe hearing loss in workers of every country in the world. The workers in industries like mining, construction, printing, saw mills, crushers, etc are at risk. Workers are exposed to high levels of noise throughout their lifetime of work, but there are very few NIHL studies in India to show its prevalence.

Awareness should be created among workers about the harmful effects of noise on hearing and other body systems by implementing education and training programs.

Research studies are needed to know the exact prevalence of NIHL among various industries in India.

A national program should be established considering the amount of damage the NIHL causes to the quality of life of workers.
